# High‐resolution genotyping indicates that children with type 1 diabetes and celiac disease share three HLA class II loci in DRB3, DRB4 and DRB5 genes

**DOI:** 10.1111/tan.14105

**Published:** 2020-11-09

**Authors:** Shehab Alshiekh, Marlena Maziarz, Daniel E. Geraghty, Helena E. Larsson, Daniel Agardh

**Affiliations:** ^1^ Department of Clinical Sciences, Lund University/CRC Skåne University Hospital SUS Malmö Sweden; ^2^ Department of Medicine King Abdulaziz University Jeddah Saudi Arabia; ^3^ Clinical Research Division Fred Hutchinson Cancer Research Center Seattle Washington USA

**Keywords:** celiac disease, children, HLA, next‐generation sequencing, type 1 diabetes

## Abstract

Type 1 diabetes (T1D) and celiac disease (CD) share common genetic loci, mainly within the human leukocyte antigen (HLA) class II complex. Extended genotyping of HLA class II alleles and their potential risk for developing both diseases remains to be studied. The present study compared extended HLA‐class II gene polymorphisms in children with T1D, CD, and a subgroup diagnosed with both diseases (T1D w/CD).

Next‐generation targeted sequencing (NGTS) of HLA‐DRB3, DRB4, DRB5, DRB1, DQA1, DQB1, DPA1, and DPB1 alleles from DNA collected from 68 T1D, 219 CD, and seven T1D w/CD patients were compared with 636 HLA‐genotyped Swedish children from the general population selected as controls.

In comparison to controls, the DRB4*01:03:01 allele occurred more frequently in T1D w/CD (odds ratio (OR) = 7.84; 95% confidence interval (95% CI) = (2.24, 34.5), *P* = 0.0002) and T1D (OR = 3.86; 95% CI, (2.69, 5.55), *P* = 1.07 × 10^−14^), respectively. The DRB3*01:01:02 allele occurred more frequently in CD as compared to controls (OR = 7.87; 95% CI, (6.17, 10.03), *P* = 4.24 × 10^−71^), but less frequently in T1D (OR = 2.59; 95% CI, (1.76, 3.81), *P* = 7.29 × 10^−07^) and T1D w/CD (OR = 0.87; 95% CI, (0.09, 3.96), *P* ≤ 0.999). The frequency of the DRB4*01:03:01‐DRB1*04:01:01‐DQA1*03:01:01‐DQB1*03:02:01 (DR4‐DQ8) haplotype was higher in T1D w/CD (OR = 12.88; 95% CI (4.35, 38.14) *P* = 3.75 × 10^−9^), and moderately higher in T1D (OR = 2.13; 95% CI (1.18, 3.83) *P* = 0.01) compared with controls, but comparable in CD (OR = 1.45; 95% CI (0.94, 2.21), *P* = 0.08) and controls.

Children with T1D and CD are associated with DRB4*01:03:01, DRB3*01:01:02, and DRB3*02:02:01 of which DRB4*01:03:01 confers the strongest risk allele for developing T1D w/CD.

## 1 INTRODUCTION

Type 1 diabetes (T1D) and celiac disease (CD) are two of the most common autoimmune diseases in the western world.^1^, ^2^

Estimated incidence rates of T1D continue rising by 1.4% annually,^3^ and CD prevalence is estimated at 1.4% worldwide.^4^ CD occurs in 3% to 16% of patients with previously diagnosed T1D.^5^, ^6^ Conversely, individuals with prior CD are at a 3‐fold increased risk for T1D before the age of 20.^7^ The risk of developing both diseases is thus significantly higher compared to the general population (GP), which is proposed to be partly explained by shared genetics.^8^, ^9^

Albeit both diseases are also associated with major histocompatibility complex (MHC) class I gene variants,^10^ the strongest genetic association has been attributed to specific MHC class II HLA‐DRB1, ‐DQA1 and ‐DQB1 genes that are in linkage disequilibrium.^11^ Nearly all patients with CD and T1D carry either the DRB1*03‐DQA1*05:01‐DQB1*02:01 (DR3‐DQ2.5) or DRB1*04‐DQA1*03‐DQB1*03:02 (DR4‐DQ8) haplotypes.^12^, ^13^ The distribution of these two haplotypes in different genotypes further confers different risk for T1D and CD. Individuals homozygous for the DR3‐DQ2.5 genotype are at the highest risk for developing CD, whereas DR3‐DQ2.5/DR4‐DQ8 is the high‐risk genotype for T1D.^14^ The risk of developing both T1D and CD (T1D w/CD) has previously been attributed to being homozygous for DQ2 or heterozygous for DQ2.5/DQ8.^15^, ^16^

Although the HLA‐DRB1, ‐DQA1 and ‐DQB1 genotypes mentioned above are prerequisites for CD and T1D, additional genes likely contribute to the disease risk.^16^, ^17^ To date, genome‐wide association studies (GWAS) have identified 41 non‐HLA loci associated with CD^18^ and 50 susceptibility genes associated with T1D.^19^ Of these, only three non‐HLA loci were identified as related to T1D w/CD; RGS1 on chromosome 1q31, IL18RAP on chromosome 2q12 and TAGAP on chromosome 6q25,^20^ respectively.

Since next‐generation targeted sequencing (NGTS) for extended genotyping of the HLA gene complex was developed, new associations between subtypes of DRB1, DRB3, DRB4 and DRB5 and T1D have been found.^21^ In a previous study applying NGTS, we found that the risk of TID was further modulated by having the DRB3*01:01:02 and DRB3*02:02:01 alleles, or not.^22^ When using the same methodology for full‐length HLA‐genotyping, it was later discovered that the DRB3*01:01:02 and DRB3*02:02:01 alleles also distinguished the risk for CD in DR3‐DQ2 carriers.^23^ However, studies of HLA‐DR and DQ polymorphisms that contribute to common genetic estimation for both diseases remain to be investigated. The aim of the present study was to extend the findings of our two previous investigations and perform high‐resolution genotyping using NGTS in children with T1D and CD in the search for shared extended HLA class II loci in children that develop both diseases.

## 2 MATERIALS AND METHODS

### 2.1 Study subjects

Included were children prospectively followed in a birth cohort screened for T1D and CD between 2004 and 2010 at the Unit of Diabetes and Celiac disease, Department of Clinical Sciences, Lund University, Malmö, Sweden, as described elsewhere.^24^ Two hundred nineteen children were diagnosed with CD (137 females, 82 males) at median age 4.5 (range 1.1‐11.0) years according to ESPGHAN criteria,^25^ 68 children were diagnosed with T1D (39 females, 29 males) at median age 5.5 (range 0.9‐11.3) years according to the American Diabetes Association criteria,^26^ and seven children (5 females, 2 males) were diagnosed with T1D w/CD (Table 1). Representing the general population (GP), 448 healthy Swedish children^27^ and 188 healthy children randomly selected from the LifeGene prospective cohort study were included as controls.^28^ Local ethical review board approval and parental informed consent were obtained for all the study participants.

**TABLE 1:** Demographic characteristics of the study population

	Type 1 diabetes‐only (T1D only) n = 68	Celiac disease‐only (CD only) n = 219	Type 1 diabetes with celiac disease (T1D w/CD) n = 7
Median (IQR) age at diagnosis	5.5 (0.9, 11.3)	4.5 (1.1, 11.0)	4.3 (2.6, 7.2)
Female, n (%)	39 (57.3%)	137 (62.5%)	5 (71.4%)
Male, n (%)	29 (42.6%)	82 (37.4%)	2 (28.5%)

### 2.2 HLA class II high‐resolution sequencing

DNA was extracted from either a dried blood spot punch or a small volume whole blood lysate specimen format from the study subjects. HLA‐class II allele sequencing was performed using the ScisGo HLA v4 typing kit (in collaboration with Scisco Genetics Inc., Seattle, Washington). Known haplotypes were used to phase the extended haplotypes and to predict the genotypes. Amplicon‐based 2‐stage polymerase chain reaction (PCR)‐based amplification of HLA‐class II alleles and sequencing‐by‐synthesis approach by using fluorescently labeled reversible terminator nucleotides with MiSeq v2 PE500 (Illumina, San Diego, California) technology was performed as previously described.^29^, ^30^ Briefly, the laboratory steps comprise consecutive PCR reactions with barcoding incorporated into the PCRs for individual DNA sample tracking. After assay‐specific amplification, samples were tagged with unique indexes and were pooled together and applied to the MiSeq device, where they were amplified as individual clusters and ordinarily sequenced using universal sequencing primers. Subsequently, sequences were analyzed using genetic system software to report unambiguous HLA‐class II alleles and haplotypes to patients' samples simultaneously.

### 2.3 Statistical analysis

Allelic frequency distribution analysis of HLA‐class II genes was performed using the relative predispositional effects (RPE) analysis.^30^ Crude odds ratios (ORs) and their associated 95% confidence intervals (95% CI) were calculated, and *χ* ^2^ tests and Fisher's exact tests (if any cell contained fewer than three observations) were used to test whether the frequencies of a given allele/haplotype differed between cases and noncases. The RPE method was used to identify the disease risk alleles, haplotypes or genotypes with the strongest predisposing or protective effects at each iteration. The selected alleles were then removed from the dataset, and the analysis was repeated until no risk or protective alleles were identified. Comparisons of DR and DQ allele frequencies were performed for both exons 2 and 3 of chromosome 6p21 by performing pairwise comparisons between all study groups and listed in order of increasing *P*‐value, followed by the extended haplotype and genotype frequencies. In the HLA‐DR locus, alleles of HLA‐DRB1 are in linkage disequilibrium with alleles of either HLA‐DRB3 or DRB4 or DRB5. Hence, by treating these highly dependent DR subtypes and their allelic variations as different alleles, analysis of HLA‐DRB1 and the secondary DRB3, DRB4 or DRB5 alleles by PRE method produces estimates of frequencies for all haplotypes provided that expectation numbers of corresponding haplotypes are five or more copies in both patients and control subjects. *P*‐values ≤.05 were considered statistically significant, and alleles with a low frequency (≤1%) were not shown in the analysis. The *P*‐values presented are nominal and not adjusted for multiple comparisons. Analyses were performed in R (r-project.org) version 3.6.1 and R package epiDisplay version 3.5.0.1.

## 3 RESULTS

### 3.1 CD children compared with controls

HLA‐*DRB345*, ‐*DRB1*, ‐*DQA1* and ‐*DQB1* allele frequencies in children with CD compared with controls are summarized in Table S1. The allelic distributions of the disease associated DRB alleles differed significantly between all the patient groups and the controls (Table 2 and 3). Among the total of 8 *DRB345* alleles, DRB3*01:01:02 was found in 60% of the CD children, (OR = 7.87; 95% CI (6.17, 10.03), *P* = 4.24 × 10^−71^), DRB4*01:03:01 in 32% (OR = 1.45; 95% CI (1.14, 1.84), *P* = 0.002) and DRB4*01:03:02 in only 1.4% (OR = 4.4; 95% CI (1.04, 21.29), *P* = 0.021). Likewise, DRB3*02:02:01 occurred in 4.6% which was less frequent than in controls (OR = 0.25; 95% CI (0.16, 0.41), *P* = 1.23 × 10^−9^). DRB1*03:01:01 occurred in 64% of CD children which was higher than in the controls (OR = 13.35; 95% CI (10.31, 17.3), *P* = 3.82 × 10^−105^). The DRB1*04:01:01 allele was found in 19% (OR = 2.19; 95% CI (1.62, 2.96) *P* = 1.85 × 10^−7^) and DRB1*04:04:01 found in 12% (OR = 2.79; 95% CI (1.9, 4.1), *P* = 5.66 × 10^−8^). The DRB1*08:01:01 allele had a protective effect in 1% (OR = 0.21; 95% CI (0.06, 0.58), *P* = 0.0005).

**TABLE 2:** Allele frequencies of HLA‐DRB3, DRB4 and DRB5 controls (N = 636)

DRB 345 allele	Allele frequency distribution				CD vs Controls		T1D vs Controls		T1D w/CD vs Controls	
	CD (N = 219)	T1D (N = 68)	T1D w/CD (N = 7)	Controls (N = 636)	OR (95%CI)	*P‐*value	OR (95%CI)	*P‐*value	OR (95%CI)	*P‐*value
DRB3*01:01:02	263 (60%)	45 (33.1%)	2 (14.3%)	204 (16%)	7.78 (5.98, 10.13)	4.2 × 10^−71^	2.59 (1.76, 3.81)	7 × 10^−7^	0.87 (0.09, 3.96)	≥0.999
DRB3*02:02:01	20 (4.6%)	6 (4.4%)	2 (14.3%)	202 (15.9%)	0.25 (0.16, 0.41)	1 × 10^−9^	0.24 (0.11, 0.56)	0.0003	0.88 (0.1, 4.01)	≥0.999
DRB4*01:03:01	138 (31.5%)	75 (55.1%)	10 (71.4%)	307 (24.1%)	1.45 (1.14, 1.84)	0.002	3.86 (2.69, 5.55)	1 × 10^−14^	7.84 (2.24, 34.5)	2 × 10^−4^ ^a^
DRB4*01:03:02	6 (1.4%)	1 (0.7%)	0 (0%)	4 (0.3%)	4.4 (1.04, 21.29)	0.02^a^	2.35 (0.05, 23.93)	0.399^a^	‐	‐

*Note:* The odds ratios (OR) and their associated 95% confidence intervals (95% CI) were estimated using all other alleles as the reference group for each estimate. The *P*‐values are based on a chi‐squared test.

Abbreviations: %, percentage of genotyped alleles (48 for CD, 136 for T1D, 14 for T1D w/CD and 1272 for controls); CD, celiac disease; n, number of alleles; T1D, type 1 diabetes; T1D w/CD; type 1 diabetes with celiac disease.

a Indicates *P*‐values based on Fisher's exact test.

**TABLE 3:** Allele frequencies of HLA‐DRB1

DRB 1 allele	Allele frequency distribution				CD vs Controls		T1D vs Controls		T1D w/CD vs Controls	
	CD(N = 219)	T1D (N = 68)	T1D w/CD (N = 7)	Controls (N = 636)	OR (95%CI)	*P*‐value	OR (95%CI)	*P*‐value	OR (95%CI)	*P‐*value
DRB1*03:01:01	283 (64.6%)	51 (37.5%)	4 (28.6%)	153 (12%)	13.35 (10.31, 17.3)	3.8 × 10^−105^	4.39 (2.98, 6.46)	1 × 10^−15^	2.92 (0.66, 10.29)	0.08
DRB1*04:01:01	85 (19.4%)	53 (39%)	9 (64.3%)	126 (9.9%)	2.19 (1.62, 2.96)	1.85 × 10^−7^	5.81 (3.93, 8.58)	4 × 10^−22^	16.3 (4.82, 62.92)	1.5 × 10^−06^ ^a^
DRB1*04:02:01	2 (0.5%)	5 (3.7%)	0 (0%)	7 (0.6%)	0.53 (0.06, 2.42)	0.534^a^	6.88 (1.7, 25.58)	0.003^a^	‐	‐
DRB1*04:04:01	54 (12.3%)	15 (11%)	0 (0%)	61 (4.8%)	2.79 (1.9, 4.1)	5 × 10^−8^	2.46 (1.36, 4.46)	0.002	‐	‐
DRB1*04:08:01	4 (0.9%)	9 (6.6%)	0 (0%)	53 (4.2%)	0.21 (0.06, 0.58)	0.462^a^	1.63 (0.79, 3.38)	0.185	‐	‐

*Note:* The odds ratios (OR) and their associated 95% confidence intervals (95% CI) were estimated using all other alleles as the reference group for each estimate. The *P*‐values are based on a chi‐squared test.

Abbreviations: %, percentage of genotyped alleles (438 for CD, 136 for T1D, 14 for T1D w/ CD and 1272for controls); CD, celiac disease; n, number of alleles; T1D, type 1 diabetes; T1D w/CD; type 1 diabetes with celiac disease.

a Indicates *P*‐values based on Fisher's exact test.

Among the HLA‐DQA1 and DQB1 alleles, DQA1*05:01:01 occurred in 64% of the CD children (OR = 13.35; 95% CI (10.31, 17.3), *P* = 3.82 × 10^−105^), DQA1*03:01:01 in 31% (OR = 3.09; 95% CI (2.38, 4.01), *P* = 2.92 × 10^−18^) and DQA1*03:02:01 in 2.5% (OR = 0.38; 95% CI (0.2, 0.72), *P* = 0.002). The DQB1*02:01:01 allele occurred in 54% of the CD children (OR = 14.3; 95% CI (11, 18), *P* = 2.06 × 10^−109^) and DQB1*03:02:01 in 33% (OR = 3.48; 95% CI (2.68, 4.5), *P* = 1.46 × 10^−22^). DQB1*04:02:01 occurred in 1% (OR = 0.19; 95% CI (0.05, 0.53), *P* = 0.0001).

A complete list of HLA‐DRB‐DQA1‐DQB1 haplotypes is summarized in Table S4. Of these, only two haplotypes were observed more frequently in CD children compared with controls: DRB3*01:01:02‐DRB1*03:01:01‐DQA1*05:01:01‐DQB1*02:01:01 (*DRB3‐DR3‐DQ2.5*) was found in 46% of CD children (OR = 7.78; 95% CI (5.98, 10.13), *P* = 1.12 × 10^−61^) and DRB3*02:02:01‐DRB1*03:01:01‐DQA1*05:01:01‐DQB1*02:01:01 in 3% (OR = 2.02; 95%CI (0.99,4.12), *P* = 0.0496). On a genotype level, a total of 29 different haplotype combinations were found in CD children compared with 315 in controls. Being homozygous for DRB3*01:01:02‐DRB1*03:01:01‐DQA1*05:01:01‐DQB1*02:01:01(DR3‐DQ2.5/DR3‐DQ2.5) was the most significant genotype (43) of CD group when compared to the control group (OR = 96 (38.55, 307.62), *P* = 1.42 × 10^−57^, Data not shown).

### 3.2 T1D children compared with controls

HLA‐*DRB345, ‐DRB1, ‐DQA1* and ‐*DQB1* allele frequencies in T1D children and controls are summarized in Table S2. Of these alleles, DRB4*01:03:01 was found in 55% of T1D children (OR = 3.86; 95% CI (2.69, 5.55), *P* = 1.07 × 10^−14^), DRB3*01:01:02 in 34% (OR = 2.59; 95% CI (1.76, 3.81), *P* = 7.29 × 10^−07^), DRB3*02:02:01 in 4.4%, which were less frequent compared with controls (OR = 0.24; 95%CI (0.11, 0.56), *P* = 0.0003). The DRB1*04:01:01 allele was the most frequent DRB1 allele and occurred in 39% (OR = 5.81; 95% CI (3.93, 8.58), *P* = 4 × 10^−22^), followed by DRB1*03:01:01 in 38% (OR = 4.39; 95% CI (2.98, 6.46), *P* = 1.05 × 10^−15^), and DRB1*04:04:01 in 11% (OR = 2.46; 95% CI (1.36, 4.46), *P* = 0.002). The DQA1*03:01:01 allele occurred in 52% of T1D children (OR = 7.48; 95% CI (5.15, 10.89), *P* = 5.38 × 10^−3^) and DQA1*05:01:01 in 38% (OR = 4.39; 95% CI (2.98, 6.46), *P* = 1.05 × 10^−15^). DQB1*03:02:01 was present in 54% (OR = 8.3; 95% CI (5.7, 12.08), *P* = 1.29 × 10^−35^) and DQB1*02:01:01 in 37.5% (OR = 4.7; 95% CI (3.19, 6.93), *P* = 4.43 × 10^−17^). DPB1*04:02:01 occured in 2.2%, which was less frequent compared with controls (OR = 0.17; 95% CI (0.03, 0.15), *P* = 0001). In contrast, DPB1*01:01:01 was associated with increased risk (OR = 2.51; 95% CI (1.45, 4.34), *P* = 0007).

Haplotype frequencies in T1D children are summarized in Table S5. Only DRB4*01:03:01‐DRB1*04:01:01‐DQA1*03:01:01‐DQB1*03:02:01 (*DRB4‐DR4‐DQ8*) was more frequently found in T1D children (11%) compared with controls (OR = 2.13; 95% CI (1.18, 3.83), *P* = 0.01). On a genotype level, 20 different different haplotype combinations were identified. Among those, DRB3*01:01:02‐DRB1*03:01:01‐DQA1*05:01:01‐DQB1*02:01:01(*DR3‐DQ2.5/DR3‐DQ2.5*) was found in 4.4% of T1D children (OR = 5.8; 95% CI (0.88, 30.6), *P* = 0.0339, Data not shown).

### 3.3 T1D w/CD children compared with controls

HLA class II alleles frequencies are summarized in Table S3. DRB4*01:03:01 was the most frequent occurring allele and found in 71.4% of T1D w/CD children (OR = 7.84; 95% CI (2.24, 34.5), *P* = 0.0002), in comparison to 14.3% that carried either DRB3*01:01:02 or DRB3*02:02:01(*P* ≤ 0.999). DRB1*04:01:01 was present in 64% of T1D w/CD (OR = 16.3; 95% CI (4.82, 62.92), *P* = 1.53 × 10^−06^), DRB1*03:01:01 in 29% (OR = 2.92; 95% CI (0.66, 10.29), *P* = 0.08). DQA1*03:01:01 was present in 57% of T1D w/CD children (OR = 9.14; 95% CI (3.13, 26.67), *P* = 1.07 × 10^−06^) and DQA1*05:01:01 in 29% (OR = 2.92; 95% CI (0.66, 10.29, *P* = 0.08). DQB1*03:02:01 occurred in 71% of T1D w/CD children (OR = 17.3; 95% CI (4.92, 76.47), *P* = 7.95 × 10^−07^) and DQB1*02:01:01 in 29% (OR = 3.13; 95% CI (0.71, 11.02), *P* = 0.06). Haplotype distributions are listed in Table S6. Among those, only DRB4*01:03:01‐DRB1*04:01:01‐DQA1*03:01:01‐DQB1*03:02:01 occurred more frequently in T1D w/CD children compared with controls and found in 42.9% (OR = 12.88; 95% CI (4.35, 38.14), *P* = 3.75 × 10^−9^). Four different *DRB3 DRB4 DRB5‐DRB1‐DQA1‐DQB1* genotypes were found among T1D w/CD children, but there was no difference compared with controls.

### 3.4 Inter‐group comparisons

By estimating the hierarchy of risk among all ‐*DRB3*, *‐DRB4, ‐DRB5* alleles using RPE analysis (Table S1‐S2‐S3), DRB4*01:03:01 rank higher in T1D and T1D w/CD predisposition than DRB3*01:01:02, which rank higher in CD predisposition in comparison to the control group. DRB4*01:03:01 occurred in 55% of T1D children (OR = 2.58; 95% CI (1.74, 3.83), P  = 1.59 × 10^−06^) and 71% of T1D w/ CD children (OR = 5.41; 95% CI (1.53, 24.06), *P* = 0.003), which was higher compared with 32% of CD children. The opposite was true for DRB3*01:01:02, which was found in 60% of CD children compared with 33% of T1D children (OR = 0.31; 95% CI (0.21, 0.47), *P* = 7.17 × 10^−09^) (Table 2). DRB1*04:01:01 was found in 39% of T1D children (OR = 2.58; 95% CI (1.7, 3.92), *P* = 6.3 × 10^‐06^) and 64% of T1D w/CD children (OR = 7.43; 95% CI (2.17, 28.98), *P* = 0.0004) compared with only 19% of CD children, whereas DRB1*03:01:01 was found in 66% of CD children compared with 37% in T1D (OR = 0.31; 95% CI (0.21, 0.46), *P* = 3.28 × 10^−09^) and 28% of T1D w/CD (OR = 0.22; 95% CI (0.05, 0.78), *P* = 0.009), respectively (Table 3). DQB1*02:01:01 was found in 60% of CD compared with 37% of T1D (OR = 0.31; 95% CI (0.21, 0.46), *P* = 3.28 × 10^−09^) and 28% of T1D w/CD (OR = 0.22; 95% CI (0.05, 0.78), *P* = 0.009), respectively. In contrast, DQB1*03:02:01 was more frequently found in T1D (54%) but not compared with CD (34%) (OR = 2.31; 95% CI (1.56, 3.41), *P* = 2.36 × 10^−05^) compared with 71% of T1D w/CD (OR = 4.98; 95% CI (1.41, 22.13), *P* = 0.007).

These findings were also investigated in the context of risk haplotypes. DRB3*01:01:02‐DRB1*03:01:01‐DQA1*05:01:01‐DQB1*02:01:01 (*DRB3‐DR3‐DQ2.5*) was the most common haplotype and occurred in 45% of CD children compared with 5% in T1D (*P* = 5.89 × 10^−18^), while DRB3*01:01:02‐DRB1*03:01:01‐DQA1*03:01:01‐DQB1*02:01:01 was the most common haplotype in T1D. The DRB4*01:03:01‐DRB1*04:01:01‐DQA1*03:01:01‐DQB1*03:02:01 (DR4‐DQ8) haplotype was carried in 42% of T1D w/CD children compared with 11% in T1D (OR = 6.05; 95% CI (1.85, 19.82), *P* = 0.001) and 7.8% in CD (OR = 8.91; 95% CI (8.91 (2.92, 27.17), *P* = 5.33 × 10^−06^) children (Table 4).

**TABLE 4:** HLA‐DRB3, DRB4, DRB5, DRB1, DQA1 and DQB1 haplotype frequencies

HLA‐DR‐DQ haplotype				Haplotype frequency distribution				CD vs Controls		T1D vs Controls		T1D w/CD vs Controls	
DRB345	DRB1	DQA1	DQB1	CD, n (%) (n = 438)	T1D, n (%) (n = 136)	T1D w/CD, n (%) (N = 14)	Controls n (%) (n = 1272	OR (95%CI)	*P*‐value	OR (95%CI)	*P*‐value	OR (95%CI)	*P*‐value
DRB3*01:01:02	DRB1*03:01:01	DQA1*05:01:01	DQB1*02:01:01	201 (45.9%)	7 (5.1%)	0 (0%)	125 (9.8%)	7.78 (5.98, 10.13)	1 × 10^−61^	0.5 (0.23, 1.09)	0.0751	‐	‐
DRB3*02:02:01	DRB1*03:01:01	DQA1*05:01:01	DQB1*02:01:01	13 (3%)	1 (0.7%)	0 (0%)	19 (1.5%)	2.02 (0.99, 4.12)	0.0496	0.49 (0.01, 3.12)	0.713	‐	‐
DRB4*01:03:01	DRB1*04:01:01	DQA1*03:01:01	DQB1*03:02:01	34 (7.8%)	15 (11%)	6 (42.9%)	70 (5.5%)	1.45 (0.94, 2.21)	0.0879	2.13 (1.18, 3.83)	0.01	12.88 (4.35, 38.14)	3.7 × 10^−09^
DRB4*01:03:01	DRB1*04:02:01	DQA1*03:01:01	DQB1*03:02:01	1 (0.2%)	2 (1.5%)	0 (0%)	6 (0.5%)	0.48 (0.01, 4)	0.686	3.15 (0.31, 17.81)	0.176	‐	‐
DRB4*01:03:01	DRB1*04:04:01	DQA1*03:01:01	DQB1*03:02:01	23 (5.3%)	4 (2.9%)	0 (0%)	59 (4.6%)	1.14 (0.69, 1.87)	0.605	0.62 (0.16, 1.72)	0.512	‐	‐

*Note:* The OR and their associated 95% CI were estimated using all other haplotypes as the reference group for each estimate.

Abbreviations: %, percentage of genotyped haplotypes (438 for CD, 136 for T1D, 14 for T1D w/ CD and 1272 for controls); CD, celiac disease; n, number of haplotypes; T1D, type 1 diabetes; T1D w/CD; type 1 diabetes with celiac disease.

In CD children, the DRB3*01:01:02‐DRB1*03:01:01‐DQA1*05:01:01‐DQB1*02:01:01(DR3‐DQ2.5/DR3‐DQ2.5) was the most frequent genotype and found in 43% of CD children compared with 4.4% in T1D (OR = 0.06; 95% CI (0.01, 0.19), *P* = 7.92 × 10^−11^, Data not shown). In T1D, the DRB3*01:01:02‐DRB1*03:01:01‐DQA1*03:01:01‐DQB1*02:01:01/DRB4*01:03:01‐DRB1*04:01:01‐DQA1*05:01:01‐DQB1*03:02:01 (DR3‐DQ2/DR4‐DQ8) genotype was found in 38% of T1D children compared with 17% of CD children (OR = 3.05; 95% CI (1.67, 5.57), *P* = 0.0002) and 28% of children with T1D w/CD (*P* = 0.347, Data not shown). In T1D w/CD, being homozygous for DRB4*01:03:01‐DRB1*04:01:01‐DQA1*05:01:01‐DQB1*03:02:01 (DR4‐DQ8/DR4‐DQ8) was the most frequent genotype and found in 42% (OR = 21.67; 95% CI (2.68, 158.4), *P* = 0.002) compared with 3.2% in CD and 10% in T1D (OR = 6.28; 95% CI (0.76, 46.47), *P* = 0.04, Data not shown) children, respectively.

## 4 DISCUSSION

The present study aimed to fill the gap of knowledge of why some children with either T1D or CD are at an increased risk for developing both diseases (T1D w/CD) by analyzing extended HLA class II genes. The main findings were that the DRB4*01:03:01, DRB3*01:01:02, and DRB3*02:02:01 alleles, were found to be associated with T1D w/CD. Of these three alleles DRB4*01:03:01 was associated with T1D only, DRB3*01:01:02 was associated with CD only, but inversely associated with T1D only. The DRB3*02:02:01 allele was associated with a low predisposition in all three groups. The novelty of the study lies in the fact that several alleles in DRB3, DRB4 and DRB5 among T1D w/CD children seem to have an extended HLA polymorphism more similar to that in children with T1D than that in children with CD.

Dissecting the extended DRB1‐DRB3‐DRB4‐DRB5 haplotypes, DRB4*01:03:01‐containing haplotypes conferred a positive association with T1D w/CD as well as T1D in comparison to DRB3*01:01:02‐containing haplotypes that were positively associated with CD. Around 50% of T1D and 70% of T1D w/CD haplotypes carry the former allele compared with 60% of CD haplotypes which carry the latter allele. Noting that all copies of chromosome 6 have a DRB1 locus, and most, but not all, have a functional second DRB locus, DRB4 was shown to be secondary for DRB1*04 haplotypes. The increase of DRB4*01:03:01 on DRB1*04:01 haplotypes in DRB1*04:01/*04:01 T1D w/CD case subjects versus DRB1*03:01/DRB1*04:01 case subjects and control subjects could reflect linkage of disequilibrium (LD) with alleles at other high‐risk loci. The analyses of the extended DRB1‐DRB3‐DRB4‐DRB5 haplotypes suggests that the risk for developing both diseases likely resembles T1D risk.

On a genotype level, differences between T1D w/CD and T1D children were found of whom T1D w/CD children were more likely to be homozygous for DR4‐DQ8/DR4‐DQ8 compared with T1D children. In line with other studies, the DR3‐DQ2 haplotype occurred in over a third of T1D children,^30^ and as previously showed, DR3‐DQ2/DR4‐DQ8 was the most frequent genotype.^31^ Moreover, HLA‐DQ2.5 homozygosity was more common among CD children compared with T1D w/CD and T1D children, confirming the HLA dosage effect of DR3‐DQ2 on the risk of CD.^32^ These results are all in line with a previous Norwegian study,^33^ and a study conducted on Dutch patients,^10^ which showed that the T1D risk heterozygous genotype (DQ2.5/DQ8) provided a comparable frequency with T1D w/CD. In contrast, Bakker et al^34^ reported that HLA‐DQ2.5 homozygosity is expected in 30% of the T1D w/CD group, indicating that a double dose of DQ2.5 confers the highest risk for T1D patients to develop CD as shown previously.^35^

The suggested hypothesis for the increased susceptibility to CD and T1D coexistence is the putative presence of DQ heterodimers encoded by alleles in *trans*, in addition to the DQ molecules encoded by alleles in *cis*, in linkage disequilibrium; DQA1*05, and DQB1*02, encoding the DQ2.5 molecule, and DQA1*03 and DQB1*03, encoding the DQ8 molecule.^36^ Consequently, the associations of these two DR‐DQ genotypes (DR3‐DQ2.5/DR3‐DQ2.5 and DR3‐DQ2/DR4‐DQ8) with CD and T1D indicate that the mechanism of autoimmune susceptibility may partly be overlapping.

The strengths of the present study were the use of high‐resolution NGTS for extended HLA genotyping, which enabled examining the disease susceptibility between T1D w/CD and extended HLA‐DRB3, DRB4 and DRB5 alleles. The RPE analysis used to estimate the association between HLA alleles (or haplotypes) and each of the outcomes (T1D only, CD only, T1D w/CD) accounts for the fact that a high frequency of a given allele “induces” a lower frequency of all other alleles, as their total must remain constant.^37^ A limitation of the study was the small sample size for the comparison of genotype effects both within and between the three disease groups. Secondly, the study included study participants from a single site comparing children at high‐risk genotypes constituting a relatively homogeneous population with little HLA diversity. It cannot be ruled out that shared HLA loci may be different in other populations.

In conclusion, NGTS for genetic risk profiling of children with T1D and CD showed shared risk associations with DRB4*01:03:01 and DRB3*01:01:02 of which DRB4*01:03:01 conferred the strongest risk allele for developing T1D w/CD.

## CONFLICT OF INTEREST

The authors have declared no conflicting interests.

## Supporting information


**Table S1** RPE analysis of the estimated frequencies of HLA‐DRB3, DRB4, DRB5, DRB1, DQA1 and DQB1 alleles among celiac patients and population controls. (XLSX)
**Table S2** RPE analysis of the estimated frequencies of HLA‐DRB3, DRB4, DRB5, DRB1, DQA1 and DQB1 alleles among type 1 diabetes patients and population controls. (XLSX)
**Table S3** RPE analysis of the estimated frequencies of HLA‐DRB3, DRB4, DRB5, DRB1, DQA1 and DQB1 alleles among type 1 diabetes with celiac disease patients and population controls. (XLSX)
**Table S4** RPE analysis of estimated frequencies of HLA‐DRB3, DRB4, DRB5, DRB1, DQA1 and DQB1 haplotypes among celiac patients and population controls. (XLSX)
**Table S5** RPE analysis of estimated frequencies of HLA‐DRB3, DRB4, DRB5, DRB1, DQA1 and DQB1 haplotypes among type 1 diabetes and population controls. (XLSX)
**Table S6** RPE analysis of the estimated frequencies of HLA‐DRB3, DRB4, DRB5, DRB1, DQA1 and DQB1 haplotypes among type 1 diabetes with celiac disease and population controls. (XLSX)

## Data Availability

The data that support the findings of this study are available from the corresponding author upon reasonable request and approval. The dataset generated in the current study is not publicly available due to Swedish law on protecting human subjects. Still, it is available from the corresponding author and guarantor of the study (DA) upon application and approval.
