# Supplementary material for: High‐resolution genotyping indicates that children with type 1 diabetes and celiac disease share three HLA class II loci in DRB3, DRB4 and DRB5 genes
Source: HLA. 2020 Nov 9;97(1):44–51. doi: 10.1111/tan.14105 (PMC7756432; doi:10.1111/tan.14105)
Supplement: Supplementary file 1 — Table S1 RPE analysis of the estimated frequencies of HLA‐DRB3, DRB4, DRB5, DRB1, DQA1 and DQB1 alleles among celiac patients and population controls. (XLSX) Table S2 RPE analysis of the estimated frequencies of HLA‐DRB3, DRB4, DRB5, DRB1, DQA1 and DQB1 alleles among type 1 diabetes patients and population controls. (XLSX) Table S3 RPE analysis of the estimated frequencies of HLA‐DRB3, DRB4, DRB5, DRB1, DQA1 and DQB1 alleles among type 1 diabetes with celiac disease patients and population controls. (XLSX) Table S4 RPE analysis of estimated frequencies of HLA‐DRB3, DRB4, DRB5, DRB1, DQA1 and DQB1 haplotypes among celiac patients and population controls. (XLSX) Table S5 RPE analysis of estimated frequencies of HLA‐DRB3, DRB4, DRB5, DRB1, DQA1 and DQB1 haplotypes among type 1 diabetes and population controls. (XLSX) Table S6 RPE analysis of the estimated frequencies of HLA‐DRB3, DRB4, DRB5, DRB1, DQA1 and DQB1 haplotypes among type 1 diabetes with celiac disease and population controls. (XLSX) [file TAN-97-44-s001.docx]

Table S1.

Relative predispositional effects (RPE) analysis of HLA-DRB3, DRB4, DRB5, DRB1, DQA1, DQB1, DPA1, and DPB1 alleles comparing CD patients (N = 219) and population controls (N = 636). The odds ratios (OR) and their associated 95% confidence intervals (95% CI) were estimated using all other alleles as the reference group for each estimate. The p-values are based on a chi-squared test, and* indicates those based on Fisher’s exact test.

| **RPE round** | **Allele** | **CD n (%)**  **(N =219)** | **Controls n (%)**  **(N = 636)** | | **OR (95% CI)** | ***P*-value** |
| --- | --- | --- | --- | --- | --- | --- |
| 1 | DRB3*01:01:02 | 263 (60%) | 204 (16%) | 7.87 (6.17, 10.03) | | 4.2 x10^-71^ |
| 1 | DRB1*03:01:01 | 283 (64.6%) | 153 (12%) | 13.35 (10.31, 17.3) | | 3.8 x10^-105^ |
| 1 | DQA1*05:01:01 | 283 (64.6%) | 153 (12%) | 13.35 (10.31, 17.3) | | 3.8 x10^-105^ |
| 1 | DQB1*02:01:01 | 283 (64.6%) | 144 (11.3%) | 14.3 (11.01, 18.58) | | 2 x10^-109^ |
| 1 | DPA1*02:01:02 | 106 (24.2%) | 64 (5%) | 6.03 (4.32, 8.41) | | 6.2 x10^-31^ |
| 1 | DPB1*01:01:01 | 106 (24.2%) | 73 (5.7%) | 5.24 (3.8, 7.24) | | 1.3 x10^-27^ |
| 2 | DRB4*01:03:01 | 138 (78.9%) | 307 (28.7%) | 9.25 (6.28, 13.6) | | 1.3 x10^-37^ |
| 2 | DRB1*04:01:01 | 85 (54.8%) | 126 (11.3%) | 9.57 (6.63, 13.81) | | 1.3 x10^-37^ |
| 2 | DQA1*03:01:01 | 136 (87.7%) | 162 (14.5%) | 42.28 (25.44, 70.28) | | 1.1 x10^-90^ |
| 2 | DQB1*03:02:01 | 146 (94.2%) | 160 (14.2%) | 98.14 (49.05, 196.38) | | 1.8 x10^-106*^ |
| 2 | DPA1*02:02:02 | 1 (0.3%) | 34 (2.8%) | 0.1 (0, 0.63) | | 3 x10-3* |
| 2 | DPB1*03:01:01 | 54 (16.3%) | 123 (10.3%) | 1.7 (1.2, 2.4) | | 2.5x10-3 |
| 3 | DRB4*01:03:02 | 6 (16.2%) | 4 (0.5%) | 35.96 (8.08, 182.93) | | 1.2 x10^-06*^ |
| 3 | DRB1*04:04:01 | 54 (77.1%) | 61 (6.1%) | 51.57 (27.88, 95.39) | | 2.7 x10^-76^ |
| 3 | DQA1*03:02:01 | 11 (57.9%) | 81 (8.5%) | 14.87 (5.82, 38.02) | | 2.8x 10^-13^ |
| 3 | DQB1*04:02:01 | 4 (44.4%) | 58 (6%) | 12.47 (2.41, 59.67) | | 1 x10-3* |
| 4 | DRB3*02:02:01 | 20 (64.5%) | 202 (26.7%) | 5 (2.35, 10.61) | | 4.4 x10^-06^ |
| 4 | DRB1*04:02:01 | 2 (12.5%) | 7 (0.8%) | 18.62 (1.74, 110.73) | | 8.9 x10-3* |
| 4 | DQA1*04:01:01 | 3 (37.5%) | 52 (5.9%) | 9.45 (1.43, 50.18) | | 0.01* |
| 4 | DQB1**03:04:01 | 1 (20%) | 3 (0.3%) | 71.72 (1.15, 1190.3) | | 0.02* |
| 5 | DRB1*04:05:01 | 2 (14.3%) | 3 (0.3%) | 49.68 (3.83, 482.57) | | 0.002* |
| 5 | DQA1*04:01:01 | 3 (60%) | 52 (6.3%) | 22.03 (2.47, 268.13) | | 0.002* |
| 5 | DQB1*06:04:01 | 2 (50%) | 52 (5.7%) | 16.3 (1.16, 228.51) | | 0.019* |
| 6 | DRB1*08:01:01 | 4 (33.3%) | 53 (5.7%) | 8.16 (1.74, 31.65) | | 0.004* |

Abbreviations: CD, celiac disease; T1D, type 1 diabetes; n, number of alleles; %, percentage of genotyped alleles (438 for CD and 1272for controls); OR, odds ratio; 95% CI, 95% confidence interval.

Table S2.

Relative predispositional effects (RPE) analysis of HLA-DRB3, DRB4, DRB5, DRB1, DQA1, DQB1, DPA1, and DPB1 allele frequencies among T1D patients (N = 68), population controls (N = 636). The odds ratios (OR) and their associated 95% confidence intervals (95% CI) were estimated using all other alleles as the reference group for each estimate. The p-values are based on a chi-squared test, and those based on Fisher’s exact test are indicated by *.

| **RPE round** | **Allele** | **T1D n (%)**  **(N = 68)** | **Controls n (%)**  **(N = 636)** | **OR (95% CI)** | ***P*-value** |
| --- | --- | --- | --- | --- | --- |
| 1 | DRB4*01:03:01 | 75 (55.1%) | 307 (24.1%) | 3.86 (2.69, 5.55) | 1 x10^-14^ |
| 1 | DRB1*04:01:01 | 53 (39%) | 126 (9.9%) | 5.81 (3.93, 8.58) | 4 x10^-22^ |
| 1 | DQA1*03:01:01 | 71 (52.2%) | 162 (12.7%) | 7.48 (5.15, 10.89) | 5.3 x10^-32^ |
| 1 | DQB1*03:02:01 | 74 (54.4%) | 160 (12.6%) | 8.3 (5.7, 12.08) | 1.2 x10^-35^ |
| 1 | DPA1*02:01:02 | 18 (13.2%) | 64 (5%) | 2.88 (1.65, 5.02) | 0.0001 |
| 1 | DPB1*04:02:01 | 3 (2.2%) | 152 (11.9%) | 0.17 (0.03, 0.51) | 0.0001* |
| 2 | DRB3*01:01:02 | 45 (73.8%) | 204 (20.5%) | 10.91 (6.04, 19.69) | 1.8 x10^-21^ |
| 2 | DRB1*03:01:01 | 51 (61.4%) | 153 (13.4%) | 10.34 (6.44, 16.61) | 5.7x 10^-30^ |
| 2 | DQA1*05:01:01 | 51 (78.5%) | 153 (13.8%) | 22.79 (12.31, 42.17) | 7.8 x10^-41^ |
| 2 | DQB1*02:01:01 | 51 (82.3%) | 144 (12.9%) | 31.17 (15.87, 61.19) | 3.3 x10^-46^ |
| 2 | DPA1*02:01:01 | 1 (0.8%) | 70 (5.8%) | 0.14 (0, 0.82) | 0.016 * |
| 2 | DPB1*01:01:01 | 18 (13.5%) | 73 (6.5%) | 2.24 (1.29, 3.89) | 0.003 |
| 3 | DRB345*Null | 9 (56.2%) | 214 (27.1%) | 3.47 (1.28, 9.42) | 0.009 |
| 3 | DRB1*04:04:01 | 15 (46.9%) | 61 (6.1%) | 13.48 (6.43, 28.29) | 4.8 x10^-18^ |
| 3 | DQA1*04:01:01 | 8 (57.1%) | 52 (5.4%) | 23.21 (7.76, 69.35) | 1.4 x10^-15^ |
| 3 | DQB1*04:02:01 | 9 (81.8%) | 58 (6%) | 69.77 (13.98, 671.04) | 9.6 x10^-10*^ |
| 4 | DRB3*02:02:01 | 6 (85.7%) | 202 (35%) | 11.1 (1.33, 512.35) | 0.009* |
| 4 | DRB1*08:01:01 | 9 (52.9%) | 53 (5.3%) | 19.93 (7.39, 53.73) | 5.3x10^-16^ |
| 4 | DQA1*03:02:01 | 5 (83.3%) | 81 (9%) | 50.34 (5.54, 2371.99) | 3.7 x10^-05*^ |
| 4 | DQB1*03:04:01 | 1 (50%) | 3 (0.3%) | 263.87 (2.89, 16384) | 0.008* |
| 5 | DRB3*02:02:01 | 6 (85.7%) | 202 (35%) | 11.1 (1.33, 512.35) | 0.009 * |
| 5 | DRB1*04:02:01 | 5 (62.5%) | 7 (0.7%) | 206.7 (33.43, 1590.66) | 6.9 x10^-09*^ |
| 6 | DRB1*04:05:01 | 2 (66.7%) | 3 (0.3%) | 509.62 (22.1, 450359962737049) | 6.8 x10^-05*^ |

Abbreviations: T1D, type 1 diabetes; n, number of haplotypes; %, percentage of genotyped alleles (136 for T1D and 1272 for controls); OR, odds ratio; 95% CI, 95% confidence interval.

Table S3.

Relative predispositional effects (RPE) analysis of HLA-DRB3, DRB4, DRB5, DRB1, DQA1 and DQB1 allele frequencies among T1D w/ CD patients (N = 7), population controls (N = 636). The odds ratios (OR) and their associated 95% confidence intervals (95% CI) were estimated using all other alleles as the reference group for each estimate. The p-values are based on a chi-squared test, and those based on Fisher’s exact test are indicated by *.

| **RPE round** | | **Allele** | **T1D w/ CD n (%)**  **(N = 7)** | **Controls n(%)**  **(N = 636)** | **OR (95% CI)** | ***P*-value** |
| --- | --- | --- | --- | --- | --- | --- |
| 1 | DRB4*01:03:01 | | 10 (71.4%) | 307 (24.1%) | 7.84 (2.24, 34.5) | 2 x10-^4^* |
| 1 | DRB1*04:01:01 | | 9 (64.3%) | 126 (9.9%) | 16.3 (4.82, 62.92) | 1.5 x10^-06*^ |
| 1 | DQA1*03:01:01 | | 8 (57.1%) | 162 (12.7%) | 9.14 (3.13, 26.67) | 1 x10^-06^ |
| 1 | DQB1*03:02:01 | | 10 (71.4%) | 160 (12.6%) | 17.3 (4.92, 76.47) | 7.9 x10^-07*^ |
| 2 | DRB1*03:01:01 | | 4 (80%) | 153 (13.4%) | 25.8 (2.53, 1268.5) | 2 x10^-3^ * |
| 2 | DQA1*05:01:01 | | 4 (50%) | 153 (13.8%) | 6.24 (1.15, 33.85) | 0.016* |
| 3 | DQA1*03:02:01 | | 2 (50%) | 81 (8.5%) | 10.76 (0.77, 150.4) | 0.039* |

Abbreviations: T1D w/ CD; type 1 diabetes with celiac disease; n, number of alleles; %, percentage of genotyped alleles (14 for T1D w/ CD and 1272 for controls); OR, odds ratio; 95% CI, 95% confidence interval.

Table S4.

HLA-DRB3, DRB4, DRB5, DRB1, DQA1 and DQB1haplotype frequencies among CD patients (N = 219), population controls (N = 636). The odds ratios (OR) and their associated 95% confidence intervals (95% CI) were estimated using all other haplotypes as the reference group for each estimate.

| **Haplotype** | **CD n (%)**  **(n =438)** | **Controls n (%)**  **(n = 1272)** | **OR (95% CI)** | ***P*-value** |
| --- | --- | --- | --- | --- |
| DRB3*01:01:02.DRB1*03:01:01.DQA1*05:01:01.DQB1*02:01:01 | 201 (45.9%) | 125 (9.8%) | 7.78 (5.98, 10.13) | 1.1 x10^-61^ |
| DRB4*01:03:01.DRB1*04:01:01.DQA1*03:01:01.DQB1*03:02:01 | 34 (7.8%) | 70 (5.5%) | 1.45 (0.94, 2.21) | 0.087 |
| DRB3*02:02:01.DRB1*03:01:01.DQA1*05:01:01.DQB1*02:01:01 | 13 (3%) | 19 (1.5%) | 2.02 (0.99, 4.12) | 0.049 |
| DRB345*Null.DRB1*04:04:01.DQA1*03:01:01.DQB1*03:02:01 | 3 (0.7%) | 1 (0.1%) | 8.75 (0.7, 459.27) | 0.054* |
| DRB4*01:03:01.DRB1*04:02:01.DQA1*03:01:01.DQB1*03:02:01 | 1 (0.2%) | 6 (0.5%) | 0.48 (0.01, 4) | 0.686* |
| DRB4*01:03:01.DRB1*04:03:01.DQA1*03:01:01.DQB1*03:02:01 | 2 (0.5%) | 8 (0.6%) | 0.72 (0.07, 3.65) | >0.999* |
| DRB4*01:03:01.DRB1*04:04:01.DQA1*03:01:01.DQB1*03:02:01 | 23 (5.3%) | 59 (4.6%) | 1.14 (0.69, 1.87) | 0.605 |
| DRB4*01:03:02.DRB1*04:04:01.DQA1*03:01:01.DQB1*03:02:01 | 1 (0.2%) | 1 (0.1%) | 2.91 (0.04, 228.06) | 0.447* |
| DRB4*01:03:02.DRB1*04:07:01.DQA1*03:01:01.DQB1*03:02:01 | 1 (0.2%) | 1 (0.1%) | 2.91 (0.04, 228.06) | 0.447* |

Abbreviations: CD, celiac disease; n, number of haplotypes; %, percentage of genotyped haplotypes (438 for CD and 1272 for controls); OR, odds ratio; 95% CI, 95% confidence interval.

Table S5.

HLA-DRB3, DRB4, DRB5, DRB1, DQA1and DQB1 haplotype freqencies among T1D patients (N = 68), population controls (N = 636) .The odds ratios (OR) and their associated 95% confidence intervals (95% CI) were estimated using all other haplotypes as the reference group for each estimate.

| **Haplotype** | **T1D n (%)**  **(n = 136)** | **Controls n (%)**  **(n = 1272)** | **OR (95% CI)** | ***P*-value** | |
| --- | --- | --- | --- | --- | --- |
| DRB4*01:03:01.DRB1*04:01:01.DQA1*03:01:01.DQB1*03:02:01 | 15 (11%) | 70 (5.5%) | 2.13 (1.18, 3.83) | | 0.01 |
| DRB3*01:01:02.DRB1*03:01:01.DQA1*05:01:01.DQB1*02:01:01 | 7 (5.1%) | 125 (9.8%) | 0.5 (0.23, 1.09) | | 0.075 |
| DRB4*01:03:01.DRB1*04:02:01.DQA1*03:01:01.DQB1*03:02:01 | 2 (1.5%) | 6 (0.5%) | 3.15 (0.31, 17.81) | | 0.176* |
| DRB3*02:02:01.DRB1*03:01:01.DQA1*05:01:01.DQB1*02:01:01 | 1 (0.7%) | 19 (1.5%) | 0.49 (0.01, 3.12) | | 0.713* |
| DRB345*Null.DRB1*04:04:01.DQA1*03:01:01.DQB1*03:02:01 | 1 (0.7%) | 1 (0.1%) | 9.38 (0.12, 734.8) | | 0.184* |
| DRB4*01:03:01.DRB1*04:01:01.DQA1*03:01:01.DQB1*03:01:01 | 1 (0.7%) | 6 (0.5%) | 1.56 (0.03, 13.03) | | 0.510* |

Abbreviations: T1D, type 1 diabetes; n, number of haplotypes; %, percentage of genotyped haplotypes (136 for T1D and 1272 for controls); OR, odds ratio; 95% CI, 95% confidence interval.

Table S6.

HLA-DRB3, DRB4, DRB5, DRB1, DQA1 and DQB1 Haplotypes distributions frequencies among T1D w/ CD patients (N = 7), population controls (N = 636) The odds ratios (OR) and their associated 95% confidence intervals (95% CI) were estimated using all other haplotypes as the reference group for each estimate.

| **Haplotype** | **T1D w/ CD n (%)**  **(N= 7)** | **Controls n (%)**  **(N = 636)** | **OR (95% CI)** | ***P*-value** |
| --- | --- | --- | --- | --- |
| DRB4*01:03:01.DRB1*04:01:01.DQA1*03:01:01.DQB1*03:02:01 | 6 (42.9%) | 70 (5.5%) | 12.88 (4.35, 38.14) | 3.7 x10^-09^ |
| DRB3*01:01:02.DRB1*03:01:01.DQA1*03:01:01.DQB1*02:01:01 | 2 (14.3%) | 0 (0%) | - | - |
| DRB3*02:02:01.DRB1*03:01:01.DQA1*03:03:01.DQB1*02:01:01 | 2 (14.3%) | 0 (0%) | - | - |
| DRB4*01:03:01.DRB1*04:01:01.DQA1*05:01:01.DQB1*03:02:01 | 3 (21.4%) | 0 (0%) | - | - |
| DRB4*01:03:01.DRB1*04:05:01.DQA1*05:01:01.DQB1*03:02:01 | 1 (7.1%) | 0 (0%) | - | - |

Abbreviations: T1D w/ CD; type 1 diabetes with celiac disease; n, number of haplotypes; %, percentage of genotyped haplotypes (14 for T1D w/ CD and 1272 for controls); OR, odds ratio; 95% CI, 95% confidence interval.
